# The impact of monoallelic inactivation mutations in the ENPP1 gene on pediatric skeletal development: a case report and literature review

**DOI:** 10.3389/fendo.2025.1430681

**Published:** 2025-04-09

**Authors:** Siyi Lu, Ning Sun, Yuan Li, Zongyi Wu, Jingdong Zhang

**Affiliations:** Department of Pediatric Orthopedic, The Second Affiliated Hospital and Yuying Children’s Hospital of Wenzhou Medical University, Wenzhou, China

**Keywords:** ARHR2, ENPP1 gene, femoral head epiphysis slippage, osteoporosis, pediatric skeletal growth

## Abstract

**Background:**

Recently, in our clinical work, we discovered a case of abnormal bone metabolism in children resulting from an inactivated mutation of the ENPP1 gene. Through this discovery, we highlighted the impact of the ENPP1 gene on the skeletal growth and development of children, and provided new ideas for the clinical diagnosis of bone diseases in children.

**Case summary:**

A 17-year-old boy presented with abnormal gait and hip pain. The anteroposterior (AP) pelvis X-ray revealed bilateral abnormalities in the femoral metaphysis, acetabulum, and ilium bones, as well as slippage of the left femoral head epiphysis. After genetic testing was carried out, it was found that the patient had a monoallelic inactivation mutations in the ENPP1 gene, which is the pathogenic gene of Autosomal-Recessive Hypophosphatemic Rickets 2 (ARHR2). Genetic testing identified that the patient had an inactivating mutation in the ENPP1 gene, which is associated with Autosomal-Recessive Hypophosphatemic Rickets 2 (ARHR2). Since symptoms were present at the time of diagnosis, the current treatment plan includes symptomatic treatments, such as calcium supplementation and femoral epiphyseal fixation.

**Conclusion:**

We discovered that the inactivating mutation of the ENPP1 gene has an influence on bone metabolism, particularly calcium and phosphorus metabolism, which can lead to severe adverse effects on the growth and development of pediatric patients. Through this case and a review of the literature, we aim to enable clinical physicians to establish a holistic perspective during pediatric consultations.

## Introduction

Recently, we identified a patient with bilateral slipped capital femoral epiphysis, whose clinical manifestations included hip and groin pain as well as a limping gait. Genetic testing was carried out on the patient, and it was discovered that the ENPP1 gene of the patient had a monoallelic loss-of-function mutation. Slipped capital femoral epiphysis (SCFE) is the most prevalent hip disorder among adolescents, typically emerging in individuals aged 8 to 15. It is also one of the diagnoses that are most frequently overlooked in children. Clinically, it is usually presented as limping and non-specific pain in the hip, groin, thigh or knee. The occurrence of SCFE is associated with obesity, rapid growth or endocrine disorders, such as hypothyroidism, growth hormone supplementation, hypogonadism and panhypopituitarism (1). It has been reported that the inactivating mutations of the ectonucleotide pyrophosphatase/phosphodiesterase 1 (ENPP1) gene can lead to Generalized Arterial Calcification of Infancy 1 (GACI1) (OMIM #208000) and Autosomal-Recessive Hypophosphatemic Rickets 2 (ARHR2) (OMIM # 613312) (2, 3). Therefore, the study was conducted on the relationship between the bone development of the pediatric patients and the ENPP1 gene.

## Materials and result

**Figure 1:**
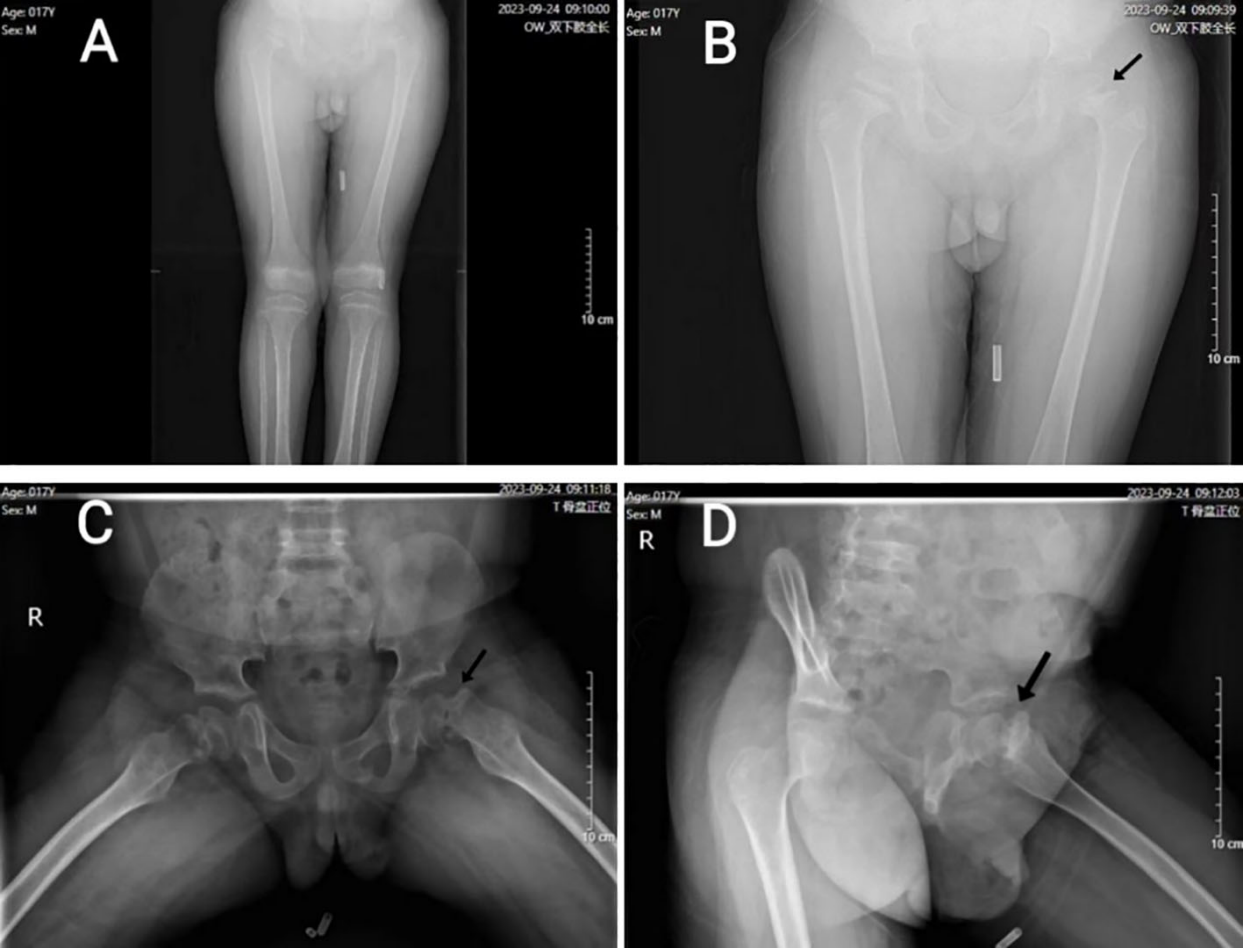
**(A)** (anteroposterior X-ray of both lower limbs): Both lower limbs were basically equal in length, and the long bones were lightly developed. **(B)** (hip and both upper limb’s anteroposterior X-ray of hip) and **(C)** (frog-like anteroposterior X-ray of hip): Bilateral femoral head epiphyseal plate widened, irregular edge; The joint surface of bilateral acetabulum was coarse, and the edge of bilateral ilium showed serrated changes. **(D)** (frog-like lateral X-ray of hip): the left femoral head was displaced medially, and the joint surface of each acetabulum was coarse; the left femoral epiphyses were separated (indicated by black arrows).

A 17-year-old boy was admitted to our hospital due to abnormal gait and hip pain. The bilateral anteroposterior X-rays of the lower limbs, along with the frog-like anteroposterior and lateral X-rays of the hip, revealed left femoral head epiphysis slip and bone abnormalities in the bilateral distal femur, tibiofibular metaphysis, acetabulum, and ilium (**Figure 1**). In addition, this patient has a one-year history of hypothyroidism and has been consistently treated with “Euthyrox”. Currently, the thyroid hormone is maintained at a normal level.

**Figure 2:**
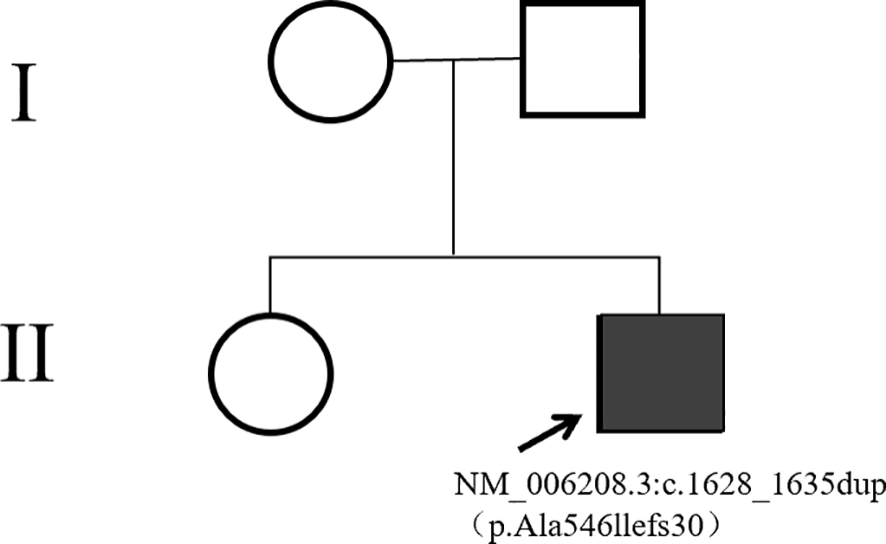
Pedigree of the family.

The patient’s height measures 127 cm (< 3^rd^ percentile for age), the weight is 38 kg, the BMI index is 23.56 (within the normal range). The result of karyotype analysis of peripheral blood is 46, XY. The peripheral blood of the patient was sent to a professional company (The King Med Diagnostics) for testing, and the result of the patient’s gene report was obtained, which clearly identified the inactivating mutation of the ENPP1 gene (**Table 1**). This mutation was a heterozygous mutation. The genes of the patient’s parents and sister were traced, and no ENPP1 gene mutation was found, and all of them were normal individuals (**Figure 2**). At the same time, the report also pointed out two genes of uncertain clinical significance (the WDR11 gene and the CDT1 gene), which clearly presented chromosomal locus variations but had not been clinically manifested. To verify the effect of the ENPP1 gene on bone metabolism, peripheral blood serum tests were also conducted for the patient (**Table 2**).

**Table 1:** Patient’s genetic report results.

Gene	Disease	Genetic mode	Chromosome position (Mutation information)	Classification of Variants
ENPP1 (Deletion mutation)	ARHR2 (OMIM # 613312)	AR	chr6:132195470_132195477 (NM_006208.3:c.1628_1635dup(p.Ala546llefs30))	P
WDR11 (missense mutation)	Hypogonadotropic hypogonadism 14 with or without anosmia, HH14 (OMIM #614858)	AD	chr10:122663620 (NM_018117.12:c.2993G>C(p.Arg998Thr))	VUS
CDT1 (missense mutation)	Meier-Gorlin syndrome 4, MGORS4 (OMIM# 613804)	AR	chr16:88870427 (NM_030928.4:c.188G>C(p.Arg63Thr))	VUS
		AR	chr16:88871904 (NM-030928.4:c.545C>T(p.Pro182Leu))	VUS

We get the report from a professional genetic company. Methods: Whole exome capture high-throughput sequencing technology. The next-generation sequencing (NGS) data obtained on the known gene exons of the human genome, and its upstream and downstream 5bp sequence of the average depth of sequencing is greater than or equal to 90X. (The DNA source used in this test is from the peripheral blood cells of the subject). Detection methods: sequencing experiment, secondary analysis, single nucleotide mutation and small fragment insertion deletion mutation analysis (SNV/Indel). Naming and interpretation of test results: Sequence variants were named according to the specifications formulated by the Human Genome Variation Society (HGVS), and classified according to *the Standards and Guidelines for the Interpretation of Sequence Variants developed* by the American College of Medical Genetics and Genomics (ACMG). Copy number variation analysis was classified according to *the Technical Standards for the Interpretation and Reporting of Constitutional Copy-Number Variants* published jointly by ACMG and Clinical Genome Resource (ClinGEN).

**Table 2:** Results of peripheral blood serum tests.

Assay Type	Specific Name	Result	Reference Value	Unit
Renal Function				
	Urea (BUN)	5.1	3.10∼8.00	mmol/L
	Creatinine (CREA)	36.9↓	57.0∼97.0	μmol/L
	Urea/Creatinine (BU/CR)	0.14	–	
	Uric Acid (UA)	433↑	208∼428	μmol/L
Thyroid Function				
	Total-triiodothyronine (TOTT3)	1.66	0.86∼1.92	ng/ml
	Total-thyroxine (TOTT4)	8.45	5.52∼11.12	μg/dl
	Free Triiodothyronine (FT3)	4.78	3.05∼4.68	μg/dl
	free thyroxine (FT4)	1.05	0.82∼1.42	pg/ml
	Thyroid-stimulating hormone (TSH)	0.894	0.51∼4.78	μIU/ml
Parathyroid Function				
	Parathyroid hormone (PTH)	25.7	15.0∼65.0	pg/ml
Growth Hormone				
	Insulin Like Growth Factor 1 (IGF1)	118.000↓	173∼414	ng/ml
	Insulin Like Growth Factor Binding Protein 3 (IGFBP3)	4.41	3.2∼8.7	μg/L
Sex Hormone				
	Androstenedione (AND)	<0.30	0.60∼3.10	ng/ml
	Dehydroepiandrosterone Sulfate (DHS)	<15.0	80.0∼560.0	μg/L
	Sex Hormone-Binding Globulin (SHBG)	22.8	10.00∼57.00	nmol/L
	Luteinizing Hormone (LH)	1.5	0.50∼5.30	IU/L
	Follicle Stimulating Hormone (FSH)	6.99	1.40∼7.50	IU/L
	Prolactin (PRL)	6.67	2.10∼17.70	ng/ml
	Progesterone (PROG)	<0.21	0.28∼1.22	ng/ml
	Estradiol(E2)	<11.80	11.6∼41.2	pg/L
	β-HCG	<1.00	0.00∼2.50	mIU/ml
Bone Metabolism				
	Bone Specific Alkaline Phosphatase (b-ALP)	92.3↑	0∼20.1	μg/L
	Procollagen Type 1 N-terminal Propeptide (P1NP)	654↑↑	15.13∼58.59	ng/ml
	Carboxy-terminal Cross-linked Telopeptide of Type 1 Collagen (CTX-1)	1722.00	1500∼2000	pg/L
	Amino-terminal Cross-linked Telopeptide of Type 1 Collagen (NTX-1)	47.20	20∼70	ng/ml
	Phosphorus (P)	1.35	0.85∼1.51	mmol/L
	Magnesium (MG)	0.79	0.75∼1.02	mmol/L
	25-Hydroxyvitamin D (1,25(OH)_2_D)	7.27↓	≥30	ng/ml

The reference values refer to the average value range of the Chinese population in this age group.

"↑" indicates exceeding the reference value, "↑↑" indicates exceeding the reference value by 5 times, and "↓" indicates being lower than the reference value.

## Discussion

In recent years, the healthy growth of children has drawn considerable attention in China, especially in terms of bone growth. SCFE and osteoporosis were diagnosed when this patient presented to the Department of Pediatric Orthopedics in our hospital. This condition is characterized by posterior and inferior displacement of the epiphysis, predominantly in the anterolateral and superior regions of the proximal femur (**Figure 1**). This situation may be associated with obesity, yet it is unrelated to any prior trauma (4). Nevertheless, the patient’s BMI is within the normal range, which suggests that other underlying factors may be responsible for the development of SCFE. The research (5) indicates that hypothyroidism can result in delayed bone ossification and epiphyseal plate hypoplasia in children. The patient had a 1-year history of hypothyroidism and was on long-term regular use of Euthyrox. Now, his clinical symptoms and thyroid hormone levels are within normal levels, and thyroid function is unaffected (**Table 2**).

Subsequently, when the patient visited in the pediatric endocrinology department, a monoallelic inactivation mutations of the ENPP1 gene was identified through genetic testing (**Table 1**).

Inactivating mutations in the ENPP1 gene can cause bone mineralization defects and renal phosphate consumption, resulting in a rare autosomal recessive form of hypophosphatemic rickets (ARHR2). It is widely accepted that this disease results from an inactivating mutation in the homozygous state of the ENPP1 gene (6). In recent years, some experts and scholars have ascertained that the inactivation mutation of a single allele of ENPP1 can likewise result in abnormal bone mineralization and disorders in phosphate metabolism. A study (7) pointed out that the heterozygous inactivated mutation of the ENPP1 gene could lead to bone mineralization defects and early-onset osteoporosis in patients. Kato et al. (8) also reported that diffuse idiopathic skeletal hyperostosis and early-onset osteoporosis are associated with haploinsufficiency of the ENPP1 gene. Bone mineralization in the human body is closely related to calcium and phosphorus metabolism, especially in pubertal children. The inactivating mutation of the ENPP1 single allele doesn’t cause death but can affect children’s growth, causing problems like delayed height development and early-onset osteoporosis.

Kotwal and colleague (9) reported that the degree of inactivating mutation of ENPP1 gene could result in the impact of gene dosage on calcium and phosphate homeostasis. The ENPP1 gene influences blood phosphorus metabolism by increasing phosphate levels and inhibiting fibroblast growth factor 23 (FGF23), thereby reducing its urinary excretion and affecting bone metabolism (10). Consequently, the inactivation of the ENPP1 gene will result in elevated FGF23 levels. Meanwhile, parathyroid hormone (PTH), 1,25-dihydroxyvitamin D3 (1,25(OH)2D3), and FGF23 collectively modulate phosphate homeostasis in the body (11, 12). In this case, the patient’s blood test (**Table 2**) showed that 1,25(OH)2D3 was significantly lower than the normal level, but the PTH level was within the normal range. Therefore, the patient’s FGF23 level might be elevated. Some researchers have also shown that an elevation in FGF23 concentration inhibits the levels of 1,25(OH)_2_D_3_ (13). At the same time, the patient’s renal function indicators were also slightly abnormal (**Table 2**). Furthermore, given that FGF23 is influenced by a multitude of factor (14), including iron levels, erythropoietin, inflammation, energy metabolism, and other metabolic parameters, it would be inappropriate to rely solely on FGF23 as an indicator of abnormal phosphorus metabolism in patients.

In addition, we examined the influence of the ENPP1 gene on bone formation and resorption. P1NP and ALP are markers for bone formation, while NTX-1 and CTX-1 indicate bone resorption (15). The patient’s bone formation markers were significantly higher than those in healthy individuals, indicating increased bone formation activity, while bone resorption remained normal. These findings suggest that the monoallelic inactivation mutations of the ENPP1 gene significantly affects bone metabolism, particularly bone formation.

The case presented in this article illustrates that the monoallelic inactivation mutations of the ENPP1 gene in the patient resulted in its inactivation, primarily manifesting as osteoporosis and bilateral slipped capital femoral epiphysis. Routine examinations, including cardiac and vascular ultrasounds, did not reveal any signs of life-threatening calcification of the aorta (16, 17), auditory impairment (18, 19), ossification of the posterior longitudinal ligament (20), or pseudoxanthoma elasticum (21–23), which are commonly associated with this gene mutation. A study (24) reported a case of ARHR2 due to biallelic pathogenic variants of ENPP1 gene. The patient exhibited abnormal gait and severe genu varum at 26 months, requiring corrective osteotomy, but it also found no other systemic diseases. At present, many clinical studies focus on the homozygous mutations of the ENPP1 gene, which can clearly lead to two clinical phenotypes: ARHR2 and GACI. Because GACI poses a significantly greater threat to patient survival, with many affected individuals succumbing during infancy (25). In contrast, ARHR2 does not directly compromise survival. As a result, older children with ENPP1 gene mutations and resultant inactivation predominantly exhibit symptoms of ARHR2, which severely impair their quality of life (26). Therefore, in many cases, the fact that the monoallelic inactivation mutations of the ENPP1 gene can also cause symptoms of ARHR2 is overlooked, which similarly imposes a significant burden on the patients’ quality of life and mental health.

Consequently, children harboring the monoallelic inactivation mutations in the ENPP1 gene might not consistently present the entire range of symptoms related to ARHR2 or be diagnosed with ARHR2. The mechanism underlying gene selective expression warrants further investigation. One limitation of our study is that we have only described the observed phenomena with conducting a literature review.

## Conclusion

In conclusion, this observation highlights the importance of considering not only lower extremity joint disorders but also endocrine and metabolic abnormalities in children presenting with an abnormal gait. Particular attention should be paid to serum indicators related to endocrine metabolism, especially during critical periods of growth and development when endocrine factors play a dominant role. It is hoped that future clinicians will expand their diagnostic scope to include potential systemic diseases beyond the immediate presenting condition, thereby promoting the overall health and well-being of pediatric patients.

## Data Availability

The original contributions presented in the study are included in the article/supplementary material, further inquiries can be directed to the corresponding author/s.

## References

[1] Peck DM Voss LM Voss TT. Slipped capital femoral epiphysis: diagnosis and management. Am Family physician. (2017) 95:779–84. 28671425

[2] Lorenz-Depiereux B Schnabel D Tiosano D Häusler G Strom TM. Loss-of-function ENPP1 mutations cause both generalized arterial calcification of infancy and autosomal-recessive hypophosphatemic rickets. Am J Hum Genet. (2010) 86:267–72. doi: 10.1016/j.ajhg.2010.01.006 PMC2820166 20137773

[3] Edouard T Linglart A. Autosomal recessive hypophosphatemic rickets type 2 due to ENPP1 deficiency (ARHR2). Arch pediatrie: organe officiel la Societe francaise pediatrie. (2024) 31:4s27–32. doi: 10.1016/s0929-693x(24)00154-4 39343470

[4] Manglunia A Goyal R Behera HB Mangaraj S. Hypothyroidism presenting as slipped capital femoral epiphysis. J paediatrics Child Health. (2022) 58:737–8. doi: 10.1111/jpc.15920 35218254

[5] Kadowaki S Hori T Matsumoto H Kanda K Ozeki M Shirakami Y. Prepubertal onset of slipped capital femoral epiphysis associated with hypothyroidism: a case report and literature review. BMC endocrine Disord. (2017) 17:59. doi: 10.1186/s12902-017-0210-6 PMC5604342 28923047

[6] Levy-Litan V Hershkovitz E Avizov L Leventhal N Bercovich D Chalifa-Caspi V. Autosomal-recessive hypophosphatemic rickets is associated with an inactivation mutation in the ENPP1 gene. Am J Hum Genet. (2010) 86:273–8. doi: 10.1016/j.ajhg.2010.01.010 PMC2820183 20137772

[7] Oheim R Zimmerman K Maulding ND Stürznickel J von Kroge S Kavanagh D. Human heterozygous ENPP1 deficiency is associated with early onset osteoporosis, a phenotype recapitulated in a mouse model of enpp1 deficiency. J Bone mineral research: Off J Am Soc Bone Mineral Res. (2020) 35:528–39. doi: 10.1002/jbmr.3911 PMC7184798 31805212

[8] Kato H Ansh AJ Lester ER Kinoshita Y Hidaka N Hoshino Y. Identification of ENPP1 haploinsufficiency in patients with diffuse idiopathic skeletal hyperostosis and early-onset osteoporosis. J Bone mineral research: Off J Am Soc Bone Mineral Res. (2022) 37:1125–35. doi: 10.1002/jbmr.4550 PMC9177665 35340077

[9] Kotwal A Ferrer A Kumar R Singh RJ Murthy V Schultz-Rogers L. Clinical and biochemical phenotypes in a family with ENPP1 mutations. J Bone mineral research: Off J Am Soc Bone Mineral Res. (2020) 35:662–70. doi: 10.1002/jbmr.3938 PMC7771569 31826312

[10] Puente-Ruiz N Docio P Unzueta MTG Lavín BA Maiztegi A Vega AI. Uncovering genetic causes of hypophosphatemia. J Internal Med. (2023) 293:753–62. doi: 10.1111/joim.13635 36999651

[11] Schaefer B Zoller H Wolf M. Risk factors for and effects of persistent and severe hypophosphatemia following ferric carboxymaltose. J Clin Endocrinol Metab. (2022) 107:1009–19. doi: 10.1210/clinem/dgab852 PMC8947794 34850000

[12] Edmonston D Wolf M. FGF23 at the crossroads of phosphate, iron economy and erythropoiesis. Nat Rev Nephrology. (2020) 16:7–19. doi: 10.1038/s41581-019-0189-5 31519999

[13] Ferreira CR Hackbarth ME Ziegler SG Pan KS Roberts MS Rosing DR. Prospective phenotyping of long-term survivors of generalized arterial calcification of infancy (GACI). Genet medicine: Off J Am Coll Med Genet. (2021) 23:396–407. doi: 10.1038/s41436-020-00983-0 PMC7867608 33005041

[14] Simic P Babitt JL. Regulation of FGF23: beyond bone. Curr osteoporosis Rep. (2021) 19:563–73. doi: 10.1007/s11914-021-00703-w PMC8958553 34757587

[15] Song L. Calcium and bone metabolism indices. Adv Clin Chem. (2017) 82:1–46. doi: 10.1016/bs.acc.2017.06.005 28939209

[16] Nitschke Y Yan Y Buers I Kintziger K Askew K Rutsch F. ENPP1-Fc prevents neointima formation in generalized arterial calcification of infancy through the generation of AMP. Exp Mol Med. (2018) 50:1–12. doi: 10.1038/s12276-018-0163-5 PMC6204430 30369595

[17] Lu P Chen J Chen M Wang L Xiang D Yin J. Case report: A rare homozygous variation in the ENPP1 gene, presenting with generalized arterial calcification of infancy in a Chinese infant. Front Cardiovasc Med. (2023) 10:1105381. doi: 10.3389/fcvm.2023.1105381 36937905 PMC10020691

[18] Brachet C Mansbach AL Clerckx A Deltenre P Heinrichs C. Hearing loss is part of the clinical picture of ENPP1 loss of function mutation. Hormone Res paediatrics. (2014) 81:63–6. doi: 10.1159/000354661 24216977

[19] Steichen-Gersdorf E Lorenz-Depiereux B Strom TM Shaw NJ. Early onset hearing loss in autosomal recessive hypophosphatemic rickets caused by loss of function mutation in ENPP1. J Pediatr Endocrinol metabolism: JPEM. (2015) 28:967–70. doi: 10.1515/jpem-2014-0531 25741938

[20] Saito T Shimizu Y Hori M Taguchi M Igarashi T Fukumoto S. A patient with hypophosphatemic rickets and ossification of posterior longitudinal ligament caused by a novel homozygous mutation in ENPP1 gene. Bone. (2011) 49:913–6. doi: 10.1016/j.bone.2011.06.029 21745613

[21] Li Q Schumacher W Jablonski D Siegel D Uitto J. Cutaneous features of pseudoxanthoma elasticum in a patient with generalized arterial calcification of infancy due to a homozygous missense mutation in the ENPP1 gene. Br J Dermatol. (2012) 166:1107–11. doi: 10.1111/j.1365-2133.2012.10811.x PMC3336007 22229486

[22] Omarjee L Nitschke Y Verschuere S Bourrat E Vignon MD Navasiolava N. Severe early-onset manifestations of pseudoxanthoma elasticum resulting from the cumulative effects of several deleterious mutations in ENPP1, ABCC6 and HBB: transient improvement in ectopic calcification with sodium thiosulfate. Br J Dermatol. (2020) 183:367–72. doi: 10.1111/bjd.18632 31646622

[23] Nitschke Y Baujat G Botschen U Wittkampf T du Moulin M Stella J. Generalized arterial calcification of infancy and pseudoxanthoma elasticum can be caused by mutations in either ENPP1 or ABCC6. Am J Hum Genet. (2012) 90:25–39. doi: 10.1016/j.ajhg.2011.11.020 22209248 PMC3257960

[24] Choe Y Shin CH Lee YA Kim MJ Lee YJ. Case report and review of literature: autosomal recessive hypophosphatemic rickets type 2 caused by a pathogenic variant in ENPP1 gene. Front endocrinology. (2022) 13:911672. doi: 10.3389/fendo.2022.911672 PMC9374118 35966073

[25] Höppner J Kornak U Sinningen K Rutsch F Oheim R Grasemann C. Autosomal recessive hypophosphatemic rickets type 2 (ARHR2) due to ENPP1-deficiency. Bone. (2021) 153:116111. doi: 10.1016/j.bone.2021.116111 34252603

[26] Ferreira CR Kintzinger K Hackbarth ME Botschen U Nitschke Y Mughal MZ. Ectopic calcification and hypophosphatemic rickets: natural history of ENPP1 and ABCC6 deficiencies. J Bone mineral research: Off J Am Soc Bone Mineral Res. (2021) 36:2193–202. doi: 10.1002/jbmr.4418 PMC8595532 34355424

